# Dietary influences on chemotherapy sensitivity and cardiotoxicity modulated by IRE1 targeting in triple‐negative breast cancer in female mice

**DOI:** 10.14814/phy2.70400

**Published:** 2025-09-21

**Authors:** Yismeilin R. Feliz‐Mosquea, Adam S. Wilson, Nildris Cruz‐Diaz, Valerie S. Payne, Katherine L. Cook, David R. Soto‐Pantoja

**Affiliations:** ^1^ Department of Physiology and Pharmacology Wake Forest University School of Medicine Winston‐Salem North Carolina USA; ^2^ Department of Surgery Wake Forest University School of Medicine Winston‐Salem North Carolina USA; ^3^ Department of Cancer Biology Wake Forest University School of Medicine Winston‐Salem North Carolina USA; ^4^ Atrium Health Wake Forest Baptist Comprehensive Cancer Center Wake Forest University School of Medicine Winston‐Salem North Carolina USA

**Keywords:** cardiac damage, doxorubicin, inositol‐requiring enzyme 1 (IRE1), lung metastases, triple‐negative breast cancer

## Abstract

Triple‐negative breast cancer (TNBC) predominantly affects young and minority women, with cytotoxic chemotherapy regimens causing severe side effects, including chronic cardiac dysfunction. Obesity worsens TNBC survival. Inositol‐requiring enzyme‐1 (IRE1), a key arm of the unfolded protein response (UPR), influences tumor progression. Using a TNBC mouse model with control and Western diets, we tested IRE1‐targeting antisense morpholino and doxorubicin. Targeting IRE1 alone reduced tumor growth and, combined with doxorubicin, did not interfere with the oncologic efficacy of this drug. We observed that increased activation of caspase‐3 was consistently activated by IRE1 in tumors regardless of diet and combination treatment. Furthermore, the blockade of IRE1 mitigated chemotherapy‐induced cardiotoxicity by preserving systolic dysfunction, reducing cardiac fibrosis, and preventing cell death. The potential difference in cell death mechanisms observed between the heart and tumors may be associated with different levels of oxidative stress as measured by 4HNE in our in vivo model. Thus, systemic IRE1 suppression protected cardiac tissue while preserving the oncologic efficacy of anthracyclines.

## 1 INTRODUCTION

Breast cancer is a multifaceted disease with various clinical and pathological features that significantly promote cancer progression, proliferation, survival, and metastasis (Feitelson et al., 2015). According to the American Cancer Society, 310,720 new breast cancer cases are predicted to occur in 2024, with more than 42,000 predictable deaths, making breast cancer the second‐highest killer of all cancer types in women in the United States (Siegel et al., 2024). Triple‐negative breast cancer (TNBC) is one of the most highly aggressive subtypes of breast cancer represents around 10%–20% of all breast cancer cases, and predominately affects young and minority women (Foulkes et al., 2010; Sorlie et al., 2003) TNBC patients are more likely to receive cytotoxic chemotherapy regimens associated with cardiac dysfunction. Increasing cumulative doses of anthracyclines are associated with cardiotoxicity and left ventricular dysfunction that, in the long term, can cause an irreversible decline in left ventricular ejection fraction in 26% of the patients (Qiu et al., 2023). It is well established that these chemotherapies cause cardiac damage. However, due to their clinical benefits, finding novel strategies to lessen the toxic side effects is essential.

Obesity is a chronic metabolic disease that represents a significant worldwide concern (Friedrich, 2017; Ng et al., 2014). Cardiovascular disease (CVD) is the leading cause of death worldwide; obesity and poor diet are a significant risk factors for CVD (Barnes, 2011; Leitner et al., 2017). Obesity is also associated with risk in 13 different types of cancers, including breast cancer. Several studies have demonstrated a strong link between obesity and increased risk of development of breast cancer, and breast cancer patients with obesity often display poor survival outcomes, especially TNBC (Biganzoli et al., 2017; Pierobon & Frankenfeld, 2013; Protani et al., 2010; Ramirez et al., 2022; Sun et al., 2017; Suzuki et al., 2009). Previous work demonstrated that a high‐fat diet promotes primary tumor growth and affects the development of doxorubicin (DOX)‐induced cardiac dysfunction by increasing fibrosis. However, the specific mechanism remains unclear (Ramirez et al., 2022). The metabolic signaling associated with obesity can activate cellular stress pathways, including the endoplasmic reticulum unfolded protein response pathway (UPR) (Fu & Doroudgar, 2022). In particular, inositol‐requiring enzyme‐1 (IRE1), an arm of the UPR, plays a crucial role in tumor development, progression, and response to therapy (Clarke et al., 2012; Clarke & Cook, 2015; Lin et al., 2008). IRE1 is mainly known for endonuclease activities; the activation results in the unconventional splicing of XBP1‐U to form the active transcription factor XBP1‐S that can lead to cancer cell proliferation and survival (Chen et al., 2014; Hu et al., 2015). However, IRE1 can also activate MAPK kinase (ASK1‐pJNK) and NFκB to promote inflammation and apoptosis, which contribute to inflammation in obesity (Soto‐Pantoja et al., 2017; Wang & Kaufman, 2012). On the other hand, the role of IRE1 signaling in cardiac injury is less clear. Conflicting reports suggest IRE1 signaling in cardiac tissue may be protective or pro‐death (Wang et al., 2018). IRE1 is a conserved regulator of the NRF2 antioxidant response (Kim, 2016). NRF2 translocates to the nucleus and subsequent ubiquitination of KEAP1 in the cytosol stimulates the transcription of cardio‐protective genes such as Hmox1 (Heme oxygenase‐1), Nqo1 (NADPH quinone oxidase‐1), Sxn1 (Sulfiredoxin‐1) and Peroxiredoxin (Steinhorn et al., 2018). We hypothesize that blockade of IRE1 signaling will preserve the oncologic efficacy of chemotherapy while preventing chemotherapy‐related cardiac toxicity in TNBC preclinical breast cancer models in the context of obesity. We show that combining IRE1‐targeting with DOX enhanced anti‐tumor chemotherapy responsiveness while protecting the heart in Western diet‐fed TNBC preclinical models.

## 2 MATERIALS AND METHODS

### 2.1 Cell culture

4T1‐luciferase‐tagged (TNBC) cells were obtained from ATCC (CRL‐1446). 4T1 cells were cultured in RPMI media supplemented with 10% FBS, penicillin/streptomycin, and glutamine, kept at 37°C and 5% CO_2_. H9C2 rat cardiac myoblast cell line was obtained from ATCC (CRL‐1446) and cultured in DMEM media supplemented with 10% FBS, penicillin/streptomycin, and glutamine, kept at 37°C and 5% CO_2_.

### 2.2 Diet composition

Prolab IsoPro RMH 3000 diet or standard diet (control diet) contains approximately 22% of proteins, 5% of fat (ether extract), 7% fat (acid hydrolysis), and 4% of fiber. The Western diet (TD. 190099) was formulated and purchased from Envigo. The Western diet contains approximately 45% kcal from fat, 18% sucrose, 7% fructose, 7% cellulose, and 3.4% NaCl. Approximate fatty acids (% total): 41.2% saturated fatty acid (SFA), 34% monounsaturated fatty acid (MUFA), and 24.8% polyunsaturated fatty acid (PUFA).

### 2.3 In vivo murine TNBC tumor model

**FIGURE 1:**
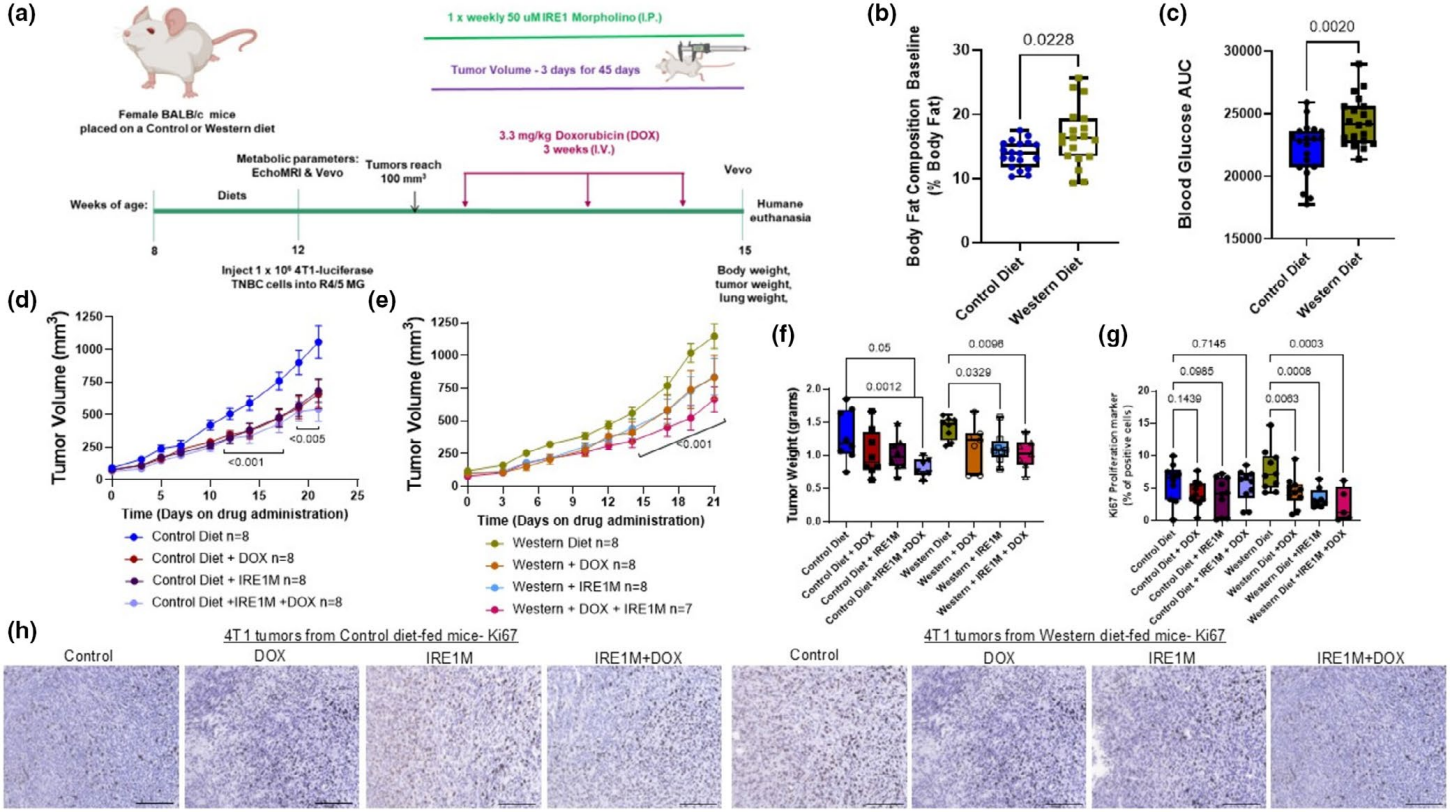
IRE1 blockade enhances the anti‐tumor effects of DOX chemotherapy in vivo. (a) In vivo treatment scheme. (b) Body fat mass composition was determined by MRI. *n* = 19–20 per diet, Unpaired *t* test. (c) Glucose area under the curve *n* = 19–20, Unpaired *t* test. (d–f) BALB/c mice were injected with 4T1 cells (1 × 10^6^ cells) into the mammary fat pad to induce tumor growth. Once tumors developed, mice were treated with DOX with or without antisense morpholino to IRE1. (d, e) Tumor volume was measured with a caliper every 3 days and was calculated by the formula: Volume = *W*2 × *L*/2, where *W* = shortest diameter and *L* = longest diameter. *n* = 7–8 per diet; differences from control treatment from control or Western diet‐fed mice were analyzed by two‐way ANOVA followed by a Tukey's multiple comparison test. (f) Tumors were excised, and weights were recorded at the end of the study. *n* = 7–8; significant differences were calculated from control treatment from control or Western diet‐fed mice; one‐way ANOVA followed by a Tukey's multiple comparison test. (g) Tumor tissue sections were stained with Ki67 as a cell proliferation marker. (h) Representative images of primary 4T1 tumors Ki67 in control and Western diet‐fed mice (scale bar = 100 μm). *n* = 6–8. IRE1M, inositol‐requiring enzyme‐1 antisense morpholino; MRI, magnetic resonance imaging.

Female 8‐week‐old BALB/c mice (*n* = 80) were purchased from Charles River (Charleston, South Carolina) and placed on a control (low‐fat, *n* = 40) or Western (high‐fat, *n* = 40) diet for 4 weeks. EchoMRI and a glucose tolerance test were performed before tumor cell inoculation. At 12 weeks of age, mice were injected with 4T1‐luciferase breast cancer cells (1 × 10^6^ cells) into the mammary fat pad. Once tumors reached an average of 100 mm^3^, mice were randomized into groups. A group received 1 × weekly 50 μM IRE1 mouse antisense morpholino (IRE1M; targets host (cardiac) and tumor) intraperitoneal (I.P.) for three subsequent weeks. Antisense morpholino (Genetools, LLC) is a molecule that inhibits the translation of the targeted protein (5′ CAGGACGTGGCCCTGACTCC 3′). Immunohistochemistry confirmed the reduction of IRE1 protein levels in tumors and hearts (see Figure S1). Groups of mismatched control (volume‐matched control) or morpholino‐treated mice received 3.3 mg/kg of doxorubicin (DOX, Sigma‐Aldrich, Cat #D1515) intravenously (I.V.) after each IRE1M injection for 3 weeks. Cardiac function was measured by VEVO LAZR ultrasound system imaging in M and B modes for systolic and diastolic cardiac outputs before cell injection and 1 week post‐last DOX injection. See Figure S2 for representative M‐mode images. Tumor growth was measured every 3 days with calipers for 21 days or until humane endpoints were reached. Tumor growth was calculated by the formula volume = *W*2 × *L*/2, where *W* = shortest diameter and *L* = longest diameter. IVIS imaging (In Vivo Imaging System®, PerkinElmer; Waltham, MA, USA) was performed weekly to monitor tumor growth and metastatic lesions in the distal organs. See the study schematic in Figure 1a. The wet weight of the tumors, lungs, and hearts was determined at the end of the study. Animals that reached humane endpoints before the 21 days were removed from the study and subjected to humane euthanasia by CO_2_ inhalation.

### 2.4 Immunohistochemistry

Tumors, lungs, and hearts were fixed in 4% paraformaldehyde for 24 h before embedding in paraffin and cut into 5 μm‐thick sections. Tumor sections were stained against IRE1α antibody (Novus Cat#, NB100‐2323; 1:500), Ki‐67 antibody (Cell Signaling Cat#, 12,202; 1:800 dilution), cleaved caspase 3 antibody (Cell Signaling Cat #9661; 1:1000 dilution) and 4HNE antibody (Abcam Cat#, ab46545; 1:400 dilution) using the Dako Envision Plus IHC staining kit (Agilent/Dako, Cat #K4065)and visualized using DAB chromogen to investigate IRE1‐targeting efficacy, tumor proliferation, apoptosis, and oxidative stress among the groups. Embedded lung tissues were stained with hematoxylin and eosin (H&E). Lung metastatic lesions were quantified, and if lesions were detected, the lesion area was measured using ImageJ software. Cross‐sectional paraffin‐embedded cardiac tissue was stained for fibrosis using a Picrosirius red pan‐collagen staining protocol (Abcam, Cat #ab245887). Also, cardiac sections were stained against IRE1α antibody (Novus Cat#, NB100‐2323; 1:500), cleaved caspase 3 antibody (Cell Signaling Cat #9661; 1:200 dilution), and 4HNE antibody (Abcam Cat#, ab46545; 1:500 dilution) using the Dako Envision Plus IHC staining kit and visualized using DAB chromogen. Staining was visualized using the Mantra Quantitative Pathology Image System with 20× and 40× objectives; 2–4 representative images from each tissue sample were quantified and averaged.

### 2.5 Viability assays

H9C2 cells were plated at a density of 10,000 per well in a 96‐well plate. H9C2 were transfected using 100 nM IRE1 morpholino. Twenty‐four hours after transfection and plating, cells were treated with 0.25 μg/mL DOX and were incubated for 24 h. Then, the cells were stained with Hoechst (Thermo‐Fischer, Cat# 62249). Cell viability was measured by fluorescent microscopy (BioTek Cytation, Agilent Technologies) or Cell Imaging Multi‐Mode Reader, combining fluorescence and high‐contrast brightfield imaging with conventional multi‐mode detection.

### 2.6 Cell respiratory measurements of H9C2 cells

The Seahorse XF96 extracellular flux analyzer was used to measure the metabolism of the H9C2 cells. Seahorse uses the mitochondrial stress test kit to measure the oxygen consumption rate or OCR, which measures mitochondrial respiration. H9C2 cells were plated at a density of 10,000 per well in a Seahorse plate. Then, H9C2 were transfected using 100 nM IRE1 morpholino. Twenty‐four hours after transfection, cells were treated with 0.25 μg/mL DOX and were incubated for 24 h. Then, the cells were treated sequentially with oligomycin, FCCP, and rotenone + antimycin A (Agilent, Cat# 103015‐100). OCR is normalized against cell counts. The measurements from these assays are expressed as OCR, and an analysis was performed using Wave software.

### 2.7 Enzyme‐linked immunosorbent assay (ELISA)

Serum was collected from the animals at the end of the study, stored at −80°C, and analyzed. The levels of B‐type natriuretic peptide or BNP (Novus Cat#, NBP2‐70011) and Troponin I type 3 (Novus Cat#, NBP3‐00456) were measured using ELISA following the manufacturer's protocol, and the plates were read immediately at 450 nm using a Bio‐Rad Benchmark Plus microplate spectrophotometer.

### 2.8 Statistics

In vivo studies were analyzed using repeated‐measures ANOVA and Brown‐Forsythe and Welch ANOVA tests followed by Tukey's multiple comparison test. The means of each condition were compared to each other. An unpaired *t* test was used to analyze the differences between two groups. Results are represented as the mean ± SD and are considered significant if *p* < 0.05. Statistical analysis was performed on GraphPad Prism.

## 3 RESULTS

### 3.1 Targeting IRE1 enhances anthracycline‐mediated tumor cytotoxicity in a murine model of TNBC

We developed a preclinical model to study chemotherapy sensitivity in the context of diet (Figure 1a). Balb/c mice were fed a control of Western diets for 4 weeks prior to tumor inoculation and continued on diet until the end of the study (Figure 1a). Mouse body fat composition was measured by nuclear magnetic resonance (NMR) using an EchoMRI™ system. Western diet consumption increased body fat mass percentage by 1.2 times compared to the control diet (13.82 ± 2.1; Control vs. 16.5 ± 4.6 Western Diet; *p* < 0.0228) (Figure 1b), establishing elevated adiposity in the diet‐induced obesity model. We also demonstrated that Western diet‐fed animals displayed elevated glucose area under the curve when compared with control diet‐fed animals after 4 weeks of diet administration (22139 ± 22082; Control vs. 24337 ± 91963 Western diet; *p* < 0.0020) (Figure 1c). In control diet‐fed animals, treatment with DOX, IRE1M, and a combination of IRE1M + DOX resulted in a significant reduction in tumor volume over time (*p* < 0.01) when compared to mismatch control animals (Figure 1d). On the other hand, tumor sensitivity to DOX was attenuated in Western diet‐fed animals, but the combination of IRE1M + DOX significantly decreased tumor volume relative to mismatch control animals on the same diet (1146 ± 267.66; Control vs. 664.9 ± 251.4; IRE1M + DOX) (Figure 1e). At the end of the study, tumors were excised, and weight was measured (Figure 1f). We observed that there was a trend suggesting that treatment with DOX reduced tumor growth compared to mismatch control in control diet‐fed animals (1.3 ± 0.38 vs. 0.4; 1.04 ± 0.35). Further analysis of tumor weight data indicated that 6 out of the 8 mice treated with DOX were 0.75 fold decreased compared to only one mouse below the mean in mismatch control mice (Figure 1f). On the other hand, a less pronounced trend was observed in Western diet‐fed mice treated with DOX (1.4 ± 0.1727; Control vs. 1.15 ± 0.3470; DOX), with only 2 out of 7 mice below the control mean. Blockade of IRE1 significantly reduced tumor weight alone or in the context of chemotherapy (1.019 ± 0.23; Control vs. 0.8039 ± 14; IRE1M) and Western diet‐fed mice (1.112 ± 0.2304; IRE1M; 1.029 ± 0.2275; IRE1M + DOX). Tumor tissue sections were stained with Ki67, a proliferation marker (Figure 1g,h). Tumors from Western diet‐fed mice had the highest number of Ki67‐positive cells compared to tumors from control diet‐fed mice. No significant differences were observed in Ki67 immunoreactivity among treatment groups of the control diet‐fed mice. However, treatment with DOX and IRE1M alone reduced Ki67 immunoreactivity by 43% and 56% (*p* < 0.05) relative to Western diet‐fed control animals. Furthermore, targeting IRE1 combined with chemotherapy reduced proliferation in the Western diet, resulting in an approximately 70% (*p* < 0.05) reduction in Ki67 immunoreactivity compared to control (Figure 1g). These results indicate that IRE1 blockade does not limit the oncologic efficacy of DOX against tumors.

### 3.2 Diet and IRE1 blockade modulate oxidative stress to enhance tumor apoptosis

**FIGURE 2:**
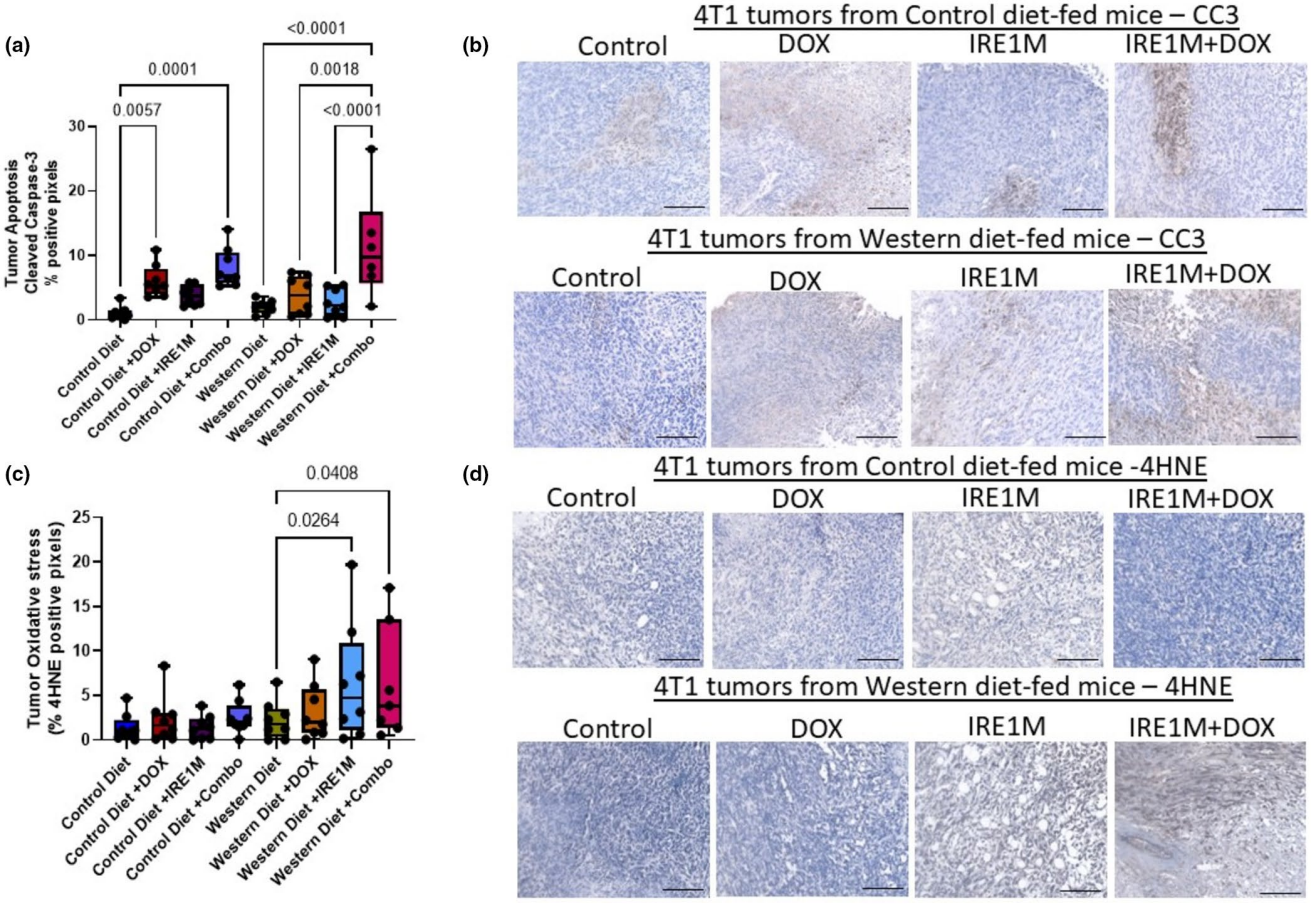
Targeting IRE1 stimulates cell death by increasing oxidative stress in tumor tissue. (a) Tumor tissue was obtained from control and Western diet‐fed mice, paraffin‐embedded, and sectioned. Sections were stained with DAB (Cleaved Caspase 3). *n* = 6–8, by two‐way ANOVA followed by a Tukey's multiple comparison test. (b) Representative image of Cleaved Caspase 3 staining 4T1 tumors from control and Western‐fed mice; *n* = 6–8. (c) Tumor oxidative stress from control and Western diet‐fed mice was measured by 4HNE staining. (d) Representative image of 4HNE staining 4T1 tumors from control and Western‐fed mice *n* = 6–8; by two‐way ANOVA followed by Tukey's multiple comparison test. DAB (3,3′‐Diaminobenzidine), 4HNE (4‐hydroxy 2‐nonenal). Scale bar = 100 μm.

Apoptosis is a programmed cell death triggered by extracellular (extrinsic apoptosis) or intracellular (intrinsic apoptosis) signals that can be measured by the induction of cleaved caspase‐3 (Suraweera et al., 2020). As expected, DOX induces cleaved caspase 3 by 5‐fold (1.074 ± 1.051; Control vs. 6.08 ± 2.48 DOX; *p* < 0.006) in the control diet. Targeting IRE1 in combination with DOX also shows a similar effect in the control diet‐fed animals (8.169 ± 3.063; IRE1M + DOX; *p* < 0.0001) (Figure 2a,b). In Western diet‐fed mice, only the combination of IRE1M + DOX induced cleaved caspase 3 by approximately 5‐fold (2.021 ± 1.026; Control vs. 11.44 ± 8.370; IRE1M + DOX; *p* < 0.0001) when compared to control Western‐fed animals (Figure 2a,b), suggesting a loss of DOX treatment efficacy in Western diet‐fed animals that can be overcome by IRE1‐targeting. Increased oxidative stress can induce apoptosis. Tumors from mice that were treated with IRE1M alone and tumors from mice treated with IRE1M + DOX showed significantly increased immunoreactivity to the oxidative stress marker 4HNE in Western diet‐fed mice (2.337 ± 2.156; Control; 6.448 ± 6.645; IRE1M; 6.306 ± 6.456; IRE1M + DOX; *p* < 0.05) (Figure 2c,d). These data suggest that the effect of targeting IRE1 on cancer cell apoptosis is associated with increased oxidative stress in Western diet‐fed mice.

### 3.3 Targeting IRE1 modulates the development of lung metastases

**FIGURE 3:**
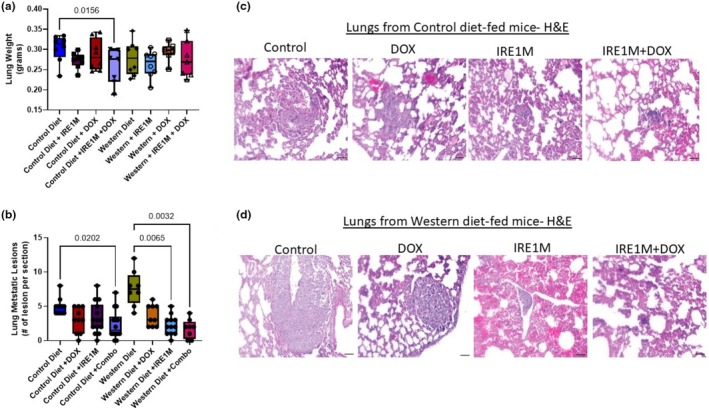
IRE1 blockade reduces lung metastasis. (a) At the end of the study, mice from the control and Western diet were euthanized, and lungs were excised and weighed. *n* = 7–8, significance was analyzed by one‐way ANOVA followed by a Tukey's multiple comparison test. (b) Lung tissue sections were paraffin‐embedded and stained with H&E to determine tissue structure and counted as the number of lesions per section *n* = 9–12; significance was analyzed by Brown‐Forsythe and Welch ANOVA tests followed by a Tukey's multiple comparison test. (c, d) Representative H&E images of lung tissue from control (c) and Western (d) diet‐fed mice. H&E, Hematoxylin and Eosin. Scale bar = 100 μm.

At the end of the study, lung weight was recorded. Targeting IRE1 in combination with DOX decreased lung weight in mice fed the control diet (0.3026 ± 0.03367; Control; 0.2563 ± 0.04349; IRE1M + DOX; *p* < 0.05) (Figure 3a). In Western diet‐fed mice, there is no difference in lung weights between treatments (Figure 3a). The lung tissues were paraffin‐embedded and stained with H&E to assess lung metastatic lesions. In mice consuming a control diet, the combination treatment of IRE1M and DOX reduces lung metastatic lesions by around 2‐fold compared to the control treatment (4.917 ± 1.165; Control vs. 2.357 ± 2.098; IRE1M + DOX) (Figure 3b,c). Targeting IRE1 alone and in combination with chemotherapy significantly reduces metastatic lesions (*p* < 0.005) when compared with control treatment in Western diet‐fed mice (7.625 ± 2.560; Control; 2.25 ± 1.422 IRE1M; 1.750 ± 1.389 IRE1M + DOX) (Figure 3b,d). These results suggest that the combination treatment of IRE1M and DOX reduces metastatic spread, which could improve the clinical outcomes of breast cancer patients.

### 3.4 IRE1 targeting preserves cardiac function after treatment with DOX

**FIGURE 4:**
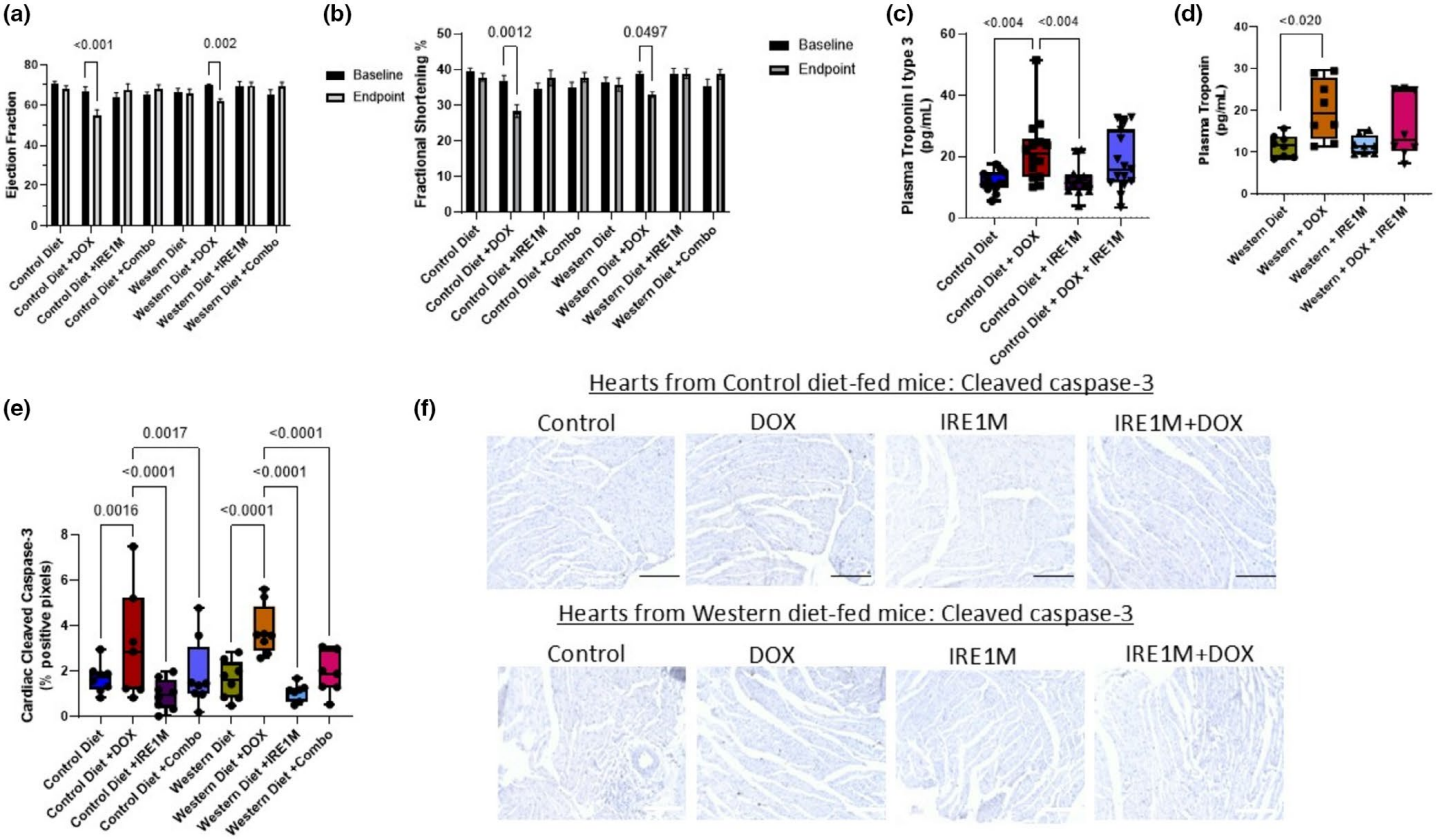
Targeting IRE1 prevents cardiac dysfunction from chemotherapy (DOX). M‐Mode tracing with in vivo ultrasound with Vevo LAZR Photoacoustic Imaging System at baseline and endpoint. Cumulative DOX dose = 10 mg/kg. (a) Ejection fraction raw data in mice consuming control and Western diets at baseline and endpoint *n* = 8. Analyzed by two‐way ANOVA followed by a Tukey's multiple comparison test. (b) Fractional shortening raw data in mice consuming control and Western diets at baseline and endpoint *n* = 8. Analyzed by two‐way ANOVA followed by Tukey's multiple comparison test. serum levels of the cardiac biomarker cTnI in Control (c) and Western (d) diets‐fed mice *n* = 8.; analyzed by two‐way ANOVA followed by Tukey's multiple comparison test. (e) Cardiac tissue was obtained from control and Western diet‐fed mice, paraffin‐embedded and sectioned. Sections were stained with DAB (Cleaved Caspase 3). *n* = 6–7, analyzed by two‐way ANOVA followed by a Tukey's multiple comparison test. (f) Representative image of Cleaved Caspase 3 staining hearts from control and Western‐fed mice (scale bar = 100 μm). cTnI, cardiac troponin I type 3; DOX, doxorubicin; I.V., intravenous.

The cardiac function was measured in mice using a VEVO LAZR ultrasound system in the M and B modes, as we have done previously (Feliz‐Mosquea et al., 2018; Ramirez et al., 2022) (representative images are shown in Figure S2A). DOX reduced left ventricular systolic function, as measured by ejection fraction (EF), from 67% at baseline to 55% at endpoint in control diet‐fed mice (*p<0.00* *1*) and from 70% at baseline to 62% (*p<0.002*) at the endpoint in Western diet‐fed mice (Figure 4a). No reduction in %EF was observed at endpoint in the context of IRE1 inhibition; furthermore, interrogation of % change of EF (baseline to endpoint change) demonstrated that blockade of IRE1 preserved EF after DOX treatment (*p* < 0.05) in mice fed control (–17.76 ±10.24; DOX vs. 6.214 ± 11.66; IRE1M + DOX) and Western diets (–10.96 ± 5.681; DOX; vs. 7.542 ± 8.532; IRE1M + DOX) when compared with DOX alone treated groups (Figure S2B). In addition, our results show that DOX treatment significantly decreased Fractional Shortening (FS) in control diet‐fed animals from 37% to 28% (p<0.0012) and from 38% to 33% (*p<0.0497*) in Western diet‐fed animals (Figure 4b). Also, significant differences were observed between DOX alone and DOX + IRE1M groups regarding the % change in FS in Western diet‐fed animals (33.029 ± 2.283; DOX vs. 38.701 ± 3.949 IRE1M + DOX) suggesting improved cardiac function using FS parameters (Figures 2b & S2B). We also measured Troponin I type 3 serum levels to assess myocardial cell wall injury. We found that DOX treatment significantly increased serum Troponin I type 3 in control (11.67 ± 3.393; Control vs. 21.91 ± 10.68; DOX) and Western (11.51 ± 2.648; Control vs. 20.22 ± 7.192; DOX) diet groups, while the combination treatment was not significantly elevated from control animals. While not significantly elevated from control, the troponin I type 3 levels of the combo‐treated groups were not different from the DOX treatment alone in both diets (Figure 4c,d). We also measured serum BNP (Figure S3) but did not observe changes in this parameter with DOX treatment in either diet. To determine whether the mechanism of cardioprotection inhibits apoptosis signaling, cross‐sectional paraffin‐embedded heart tissue was stained for cleaved caspase 3. We found that DOX significantly increased cell death marker cleaved caspase 3 by 2‐fold in control (1.680 ± 0.6471; Control vs. 3.150 ± 2.453; DOX; *p* < 0.002) and Western (1.589 ± 0.8457; Control 3.792 ± 1.094; DOX; *p* < 0.0001) diet‐fed mice (Figure 4e,f). However, IRE1 blockade decreased cleaved caspase 3 immunoreactivity in cardiac tissue when combined with chemotherapy in both diets, control (1.853 ± 1.522; IRE1 + DOX) and Western (1.867 ± 0.9275; DOX vs. IRE1M + DOX diets) (Figure 4e,f). These results suggest that targeting IRE1 with an anti‐sense morpholino could be used to prevent cardiac dysfunction in breast cancer patients receiving anthracycline chemotherapy.

### 3.5 IRE1 blockade prevents cardiac tissue remodeling associated with DOX treatment

**FIGURE 5:**
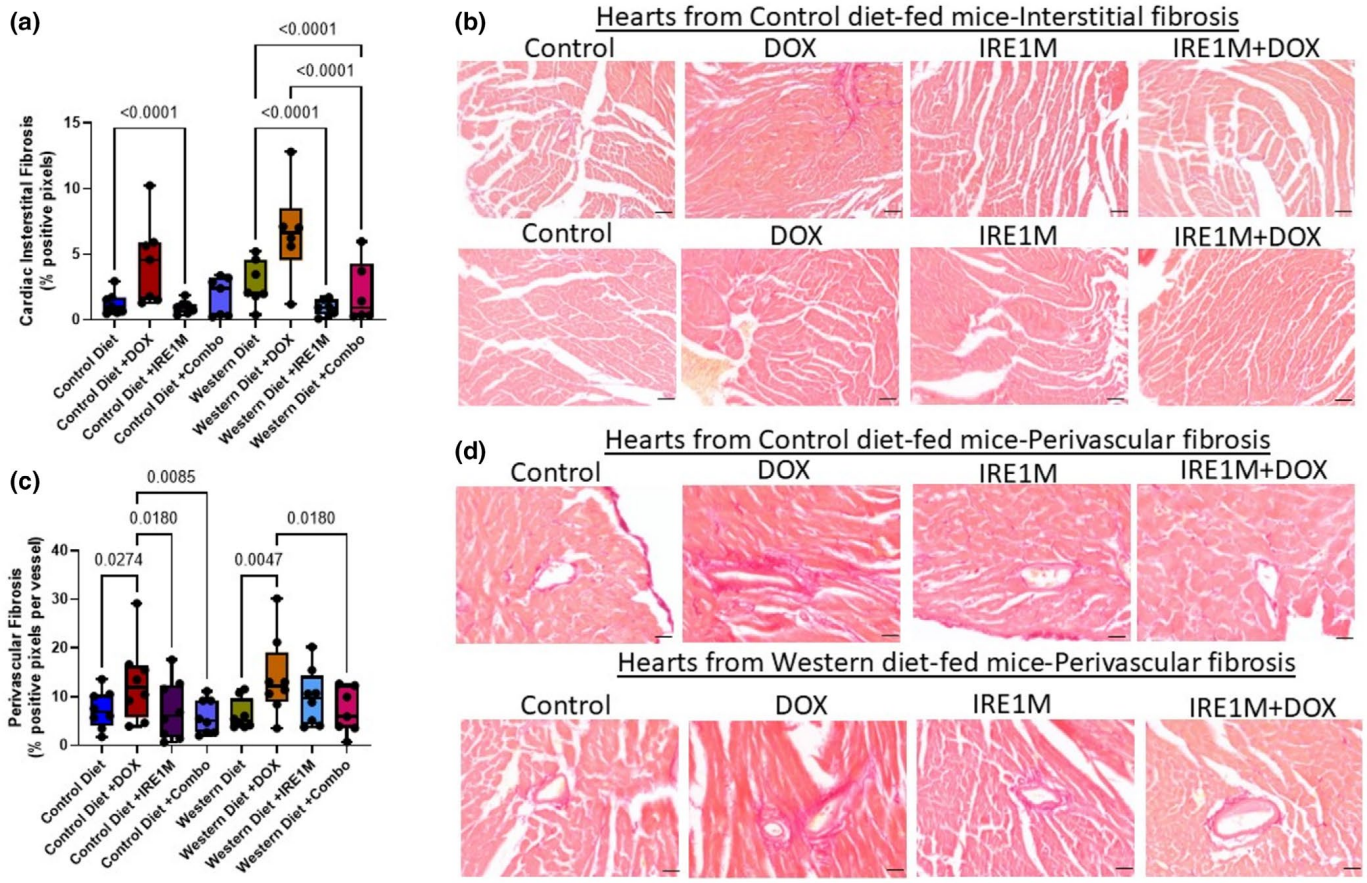
Targeting IRE1 modulates DOX‐induced fibrosis. Hearts of mice were excised after 3 weeks of IRE1M and DOX treatment, sections were paraffin‐embedded and (a–d) picrosirius red was performed. (a) Interstitial fibrosis in mice consuming control and Western diets *n* = 5–7; two‐way ANOVA followed by a Tukey's multiple comparison test. (b) Representative images of picrosirius red staining in cardiac tissue from mice on control and Western diets, indicating collagen deposition. 40×, Scale bar = 100 μm; 4 sections/animal. (c) Perivascular fibrosis in mice consuming diets. *n* = 5–7; two‐way ANOVA followed by a Tukey's multiple comparison test. (d) Representative image from control and Western diet‐fed mice. 40×, Scale bar = 100 μm; 4 sections/animal.

Cardiac fibrosis is indicative of cardiac stress and damage. Fibrosis can accumulate in cardiac tissue, limiting contractility and function. We measured cardiac fibrosis by assessing interstitial and perivascular fibrosis using Picrosirius red staining (Figure 5a–d). In Western diet‐fed mice, IRE1M + DOX treatment reduced cardiac interstitial fibrosis (Figure 5a,b) compared to DOX alone (6.660 ± 3.721; DOX vs 2.010 ± 2.346; IRE1M + DOX). Cardiac perivascular fibrosis was induced by DOX (7.368 ± 3.913 Control diet vs. 12.89 ± 8.051 DOX Control diet; and 6.303 ± 3.128 Control Western diet vs. 13.92 ± 8.210; DOX Western diet) in mice on either diet (Figure 5c,d), and this effect was ameliorated by targeting IRE1 (7.310 ± 3.521) or combination (5.908 ± 3.521) in control diet and in Western diet‐fed mice treated with the combination of IRE1M + DOX (7.019 ± 4.668). Thus, the blockade of IRE1 prevented DOX‐induced fibrotic cardiac remodeling.

### 3.6 IRE1 blockade regulated oxidative stress and cell metabolism

**FIGURE 6:**
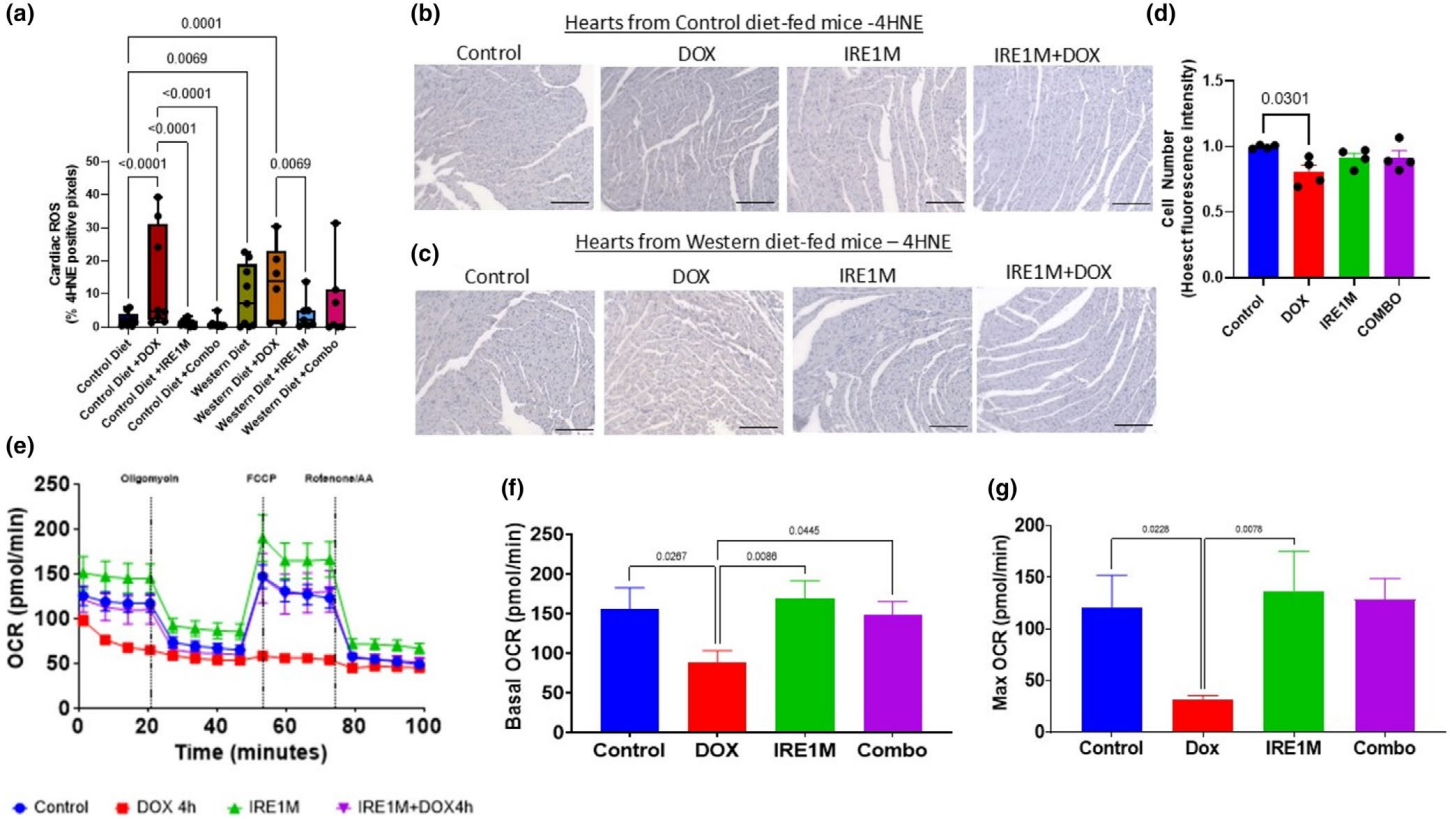
IRE1 blockade modulates mitochondria respiration. (a) Hearts oxidative stress from control and Western diets‐fed mice were measured by 4HNE staining. Representative image of 4HNE staining hearts from control (b) and Western (c) fed mice *n* = 6–8, analyzed by two‐way ANOVA followed by a Tukey's multiple comparison test (Scale bar = 100 μm). H9C2 rat cardiomyoblasts were plated and treated them with 100 nM IRE1 morpholino for 24 h. Then, cells were treated with or without 0.25 μM DOX. (d) Cell viability was measured by fluorescent microscopy (BioTek Cytation, Agilent Technologies) or Cell Imaging Multi‐Mode Reader, combining fluorescence and high contrast brightfield imaging with conventional multi‐mode detection. (e–g) Role of IRE1 in mitochondria respiration. A respirometry experiment was performed to measure the OCR which is a measure of mitochondrial respiration on H9C2 cells. (e) OCR measure, (f) basal OCR, and (g) maximal OCR. OCR was normalized to cell number; *n* = 3; two‐way ANOVA followed by a Tukey's multiple comparison test. 4HNE, 4‐hydroxy 2‐nonenal; DOX, doxorubicin; IRE1M, inositol‐requiring enzyme‐1 antisense morpholino; OCR, oxygen consumption rate.

DOX significantly increased the 4HNE oxidative stress marker compared to the control‐treated animals (2.241 ± 2.029; Control diet vs. Control diet + DOX; 14.06 ± 15.73) (Figure 6a–c). Western diet alone increased oxidative stress in cardiac tissue compared to control diet mismatch morpholino‐treated mice (9.079 ± 9.360). IRE1 blockade significantly reduced oxidative stress in heart tissue with or without combination with chemotherapy in control diet‐fed animals (1.184 ±1.042; IRE1M; 1.015 ± 1,459; IRE1M + DOX; *p* < 0.0001) when compared with DOX alone (Figure 6a–b). In Western diet‐fed animals IRE1M alone caused a reduction in cardiac oxidative stress when compared to DOX (13.50 ± 11.37; DOX vs.; 3.876 ± 4.729; IRE1M; *p* < 0.007) (Figure 6a–c). While we did not observe a significant regulation between cardiac 4HNE in control and IRE1M + DOX‐treated animals consuming the Western diet, we observed that 5 of the 6 mice had reduced 4‐HNE below the mean of the DOX + Western Diet group (Figure 6a–c). Using H9C2 rat cardiomyoblasts treated with IRE1M +/− DOX, we show that knockdown of IRE1 prevents DOX‐induced cytotoxicity in vitro (Figure 6d). Since 4‐HNE and oxidative stress can limit mitochondrial function, we performed cellular respiration analysis in H9C2 cells using a seahorse bioanalyzer. Our data show that basal respiration was reduced by approximately 50% by DOX treatment (155.2 ± 94.4; Control vs. 87.387.3 ± 54.07; DOX) (Figure 6e,f). However, treatment with DOX did not reduce basal respiration with IRE1 blockade. Furthermore, maximal respiration (calculated as basal OCR − OCR after FCCP treatment) was elevated with the DOX + IRE1 blockade compared to DOX alone (Figure 6e,g). Thus, this suggests that IRE1 blockade can preserve mitochondrial respiration and help overcome stress due to DOX treatment.

## 4 DISCUSSION

Our findings demonstrate that targeting IRE1 arm of the UPR pathway reduced tumor progression and prevented cardiac dysfunction associated with chemotherapy treatment in control and Western diet‐fed mice with TNBC tumors, representing a novel combinatorial neoadjuvant therapy regimen to protect the heart while sensitizing the tumor concurrently. IRE1 signaling can promote cell survival or cell death in response to stress. Previous studies demonstrate that in patients with TNBC, high expression of IRE1 signaling is associated with poor prognosis (Chen et al., 2014). It has also been shown that targeting IRE1 signaling can reduce tumor burden (Logue et al., 2018; Zhao et al., 2018). In our study, we used DOX, an anthracycline chemotherapeutic agent clinically effective in treating different types of cancer, especially in patients with advanced and invasive breast cancer, such as triple‐negative breast cancer. However, the limitation of DOX is that the cumulative dose can cause cardiac dysfunction that can occur either in the acute or chronic setting. Our results demonstrate that blockade of IRE1 combined with DOX reduced the tumor volume in Western diet‐fed untreated mice groups. In control diet‐fed animals, there was no significant difference in tumor volumes between the different treatments, suggesting that the blockade of IRE1 does not interfere with the oncologic efficacy of DOX. Still, our data indicate that while DOX and IRE1M + DOX treatments increased tumor apoptosis (cleaved caspase 3) in both diets, only reduced tumor proliferation (Ki67) was observed in tumors from mice consuming a Western diet in the different treatment groups. However, we did note a significant reduction in tumor weight at the end of the study in DOX + IRE1M treated mice on both diets, thereby demonstrating beneficial outcomes of the combinatorial treatment on either dietary background. Furthermore, we investigated intratumoral oxidative stress (4HNE) and found that it was significantly increased by targeting IRE1 alone or combined with DOX only in the Western diet. Overall, these results suggest that targeting IRE1 with morpholinos impaired IRE1‐mediated signaling that may disrupt the ER chaperones, ERAD, or their functions, which affect cell survival and trigger apoptosis in TNBC cells. IRE1 has been shown to have multiple functions, including unconventional splicing of XBP1 and activation of JNK, which trigger apoptosis (Glembotski, 2007; Uemura et al., 2009; Wang et al., 2020). In cancer cells, IRE1/XBP1 is a key regulator of ER stress that controls cell survival and/or apoptosis. Evidence suggests that inhibition of IRE1 (Rnase site) leads to decreased splicing of XBP1, which reduces cell proliferation and increases apoptosis in breast cancer (Ming et al., 2015), pancreatic cancer (Chien et al., 2014), and multiple myeloma (Chen et al., 2016). Consequently, IRE1 targeting is of interest for anticancer drug development strategies.

It has been demonstrated in the murine model of triple‐negative breast cancer that 4T1 cells are injected into the mammary fat pad, causing metastasis incidence to the lungs in less than 60 days (Yang et al., 2012). We found that IRE1M, in combination with chemotherapy, reduces lung lesions in the control and Western diets. However, IRE1M alone also reduces lung metastasis in the Western diet but not the control diet. These data suggest that targeting IRE1M, when combined with chemotherapy, reduces tumor progression and metastatic development.

In the United States, it is estimated that there are over 3.5 million living breast cancer survivors, but they face a more significant burden of non‐cancer chronic diseases, such as cardiovascular disease, compared to their cancer‐free peers, leading to excess morbidity and mortality, reduced quality of life, and increased medical expenditures (Sikov et al., 2015). It is well known that cumulative doses of anthracyclines cause cardiotoxicity. However, there is a gap in understanding the mechanisms of cardiotoxicity, markers of susceptibility, and complementary therapeutic strategies that ameliorate side effects without compromising oncologic efficacy. Several studies have associated cardiovascular diseases with alterations in the endoplasmic reticulum (ER), particularly disruption of ER homeostasis leading to upregulation or severe activation of the UPR, especially IRE1 (Hamada et al., 2004; Liu et al., 2008; Minamino & Kitakaze, 2010; Okada et al., 2004; Ortega et al., 2014). Activation of IRE1 has been shown to cause activation of the NLRP3/IL‐1β inflammatory pathway that is associated with inflammation in cardiovascular tissue (Hong et al., 2017). However, deletion or inhibition of inositol‐requiring enzyme 1 (IRE1) activity causes a significant reduction in cardiac injury formation by reducing caspase‐1 activity and IL‐1β secretion (Marek‐Iannucci et al., 2022), and it also attenuates cardiac fibrosis by restoring autophagy in cardiac fibroblasts (Qu et al., 2021). In conjunction with these published findings, this study investigates the effect of IRE1 blockade on cardiac function parameters, including ejection fraction and fractional shortening. We show that DOX reduced ejection fraction and fractional shortening in the control and Western diets; targeting IRE1 prevents this effect from chemotherapy in both diets. In addition, we found that IRE1 blockade reduced the cardiac dysfunction marker (Troponin I type 3) and perivascular fibrosis.

The role of the mitochondria response to DOX in generating oxidative stress and apoptosis is extensively studied (Zhu et al., 2016). DOX targets mitochondria, causing cardiomyocyte damage (Chen et al., 2020) by forming an irreversible complex with the protein cardiolipin (Goormaghtigh et al., 1983). The DOX‐cardiolipin complex causes drug accumulation in mitochondria, which disrupts the proper cardiolipin protein interface and leads to superoxide radical formation (Chen et al., 2020; Goormaghtigh et al., 1983; Schlame et al., 2000). Hence, oxidative stress from DOX changes metabolism, which can lead to heart failure or abnormal contractility and relaxation by reducing the oxidation of long‐chain fatty acids in heart mitochondria and increasing glucose metabolism (Carvalho et al., 2010; Chen et al., 2020; Kashfi et al., 1990; Raj et al., 2014; Ventura‐Clapier et al., 2004). On the other hand, several studies showed that UPR arms have a cardio‐protective effect on ischemic insults (Bi et al., 2018; Martindale et al., 2006; Petrovski et al., 2011; Thuerauf et al., 2006). Our study showed that IRE1 blockade increased mitochondrial respiration in cardiac H9C2 cells, which could explain the cardio‐protective mechanism of IRE1 blockade in our study. Our results suggest that IRE1 blockade protects from chemotherapy‐induced cardiotoxicity without reducing anti‐tumor effects and, in the context of obesity, may enhance anticancer chemotherapy efficacy.

### 4.1 Perspectives and significance

This study is consistent with the general agreement in the field that DOX is a highly effective chemotherapy; however, it could be detrimental to the long‐term quality of life of all cancer survivors due to severe cardiac dysfunction and the development of heart failure. For this reason, it is important to develop new strategies, such as targeting IRE1, that can provide better treatment benefits by reducing the side effects of chemotherapies, especially in younger patients receiving this toxic chemotherapy (DOX). Several studies investigate the cardiotoxicity effects of chemotherapy in non‐tumor‐bearing mice. We now demonstrate in a tumor model how the cardiotoxicity effect of DOX is reduced without compromising the oncological efficacy of chemotherapy by blocking IRE1.

## AUTHOR CONTRIBUTIONS

Y.R.F.‐M. data curation, formal analysis, funding acquisition, investigation, methodology, writing – original draft; D.R.S.‐P. conceptualization, supervision, funding acquisition, writing – review & editing; K.L.C. conceptualization, supervision, funding acquisition, writing – review & editing; A.S.W. project administration and methodology; N.C.‐D. and V.S.P. methodology.

## FUNDING INFORMATION

This work was supported by an American Cancer Society Research Scholar Grant RSG‐19‐150‐01‐LIB (DRS‐P), a Susan G. Komen Career Catalyst grant CCR18547795 (KLC), and the Wake Forest Baptist Comprehensive Cancer Center's NCI Cancer Center Support Grant (P30CA012197). Additionally, this work is supported by the NIH NRSA Predoctoral Fellowship in the Redox Medicine and Biology Training Program grant 5T32GM127261‐04 (YFM) through NIGMS.

## CONFLICT OF INTEREST STATEMENT

No conflicts of interest, financial or otherwise, are declared by the authors.

## ETHICS STATEMENT

The Animal Care and Use Committee of the Wake Forest University School of Medicine approved the protocol, and all procedures were carried out per relevant guidelines and regulations.

## Supporting information


Data S1.

## Data Availability

All data are presented in the article. Data is available upon request to the authors.

## References

[phy270400-bib-0001] Barnes, A. S. (2011). The epidemic of obesity and diabetes: Trends and treatments. Texas Heart Institute Journal, 38, 142–144. 21494521 PMC3066828

[phy270400-bib-0002] Bi, X. , Zhang, G. , Wang, X. , Nguyen, C. , May, H. I. , Li, X. , Al‐Hashimi, A. A. , Austin, R. C. , Gillette, T. G. , Fu, G. , Wang, Z. V. , & Hill, J. A. (2018). Endoplasmic reticulum chaperone GRP78 protects heart from ischemia/reperfusion injury through Akt activation. Circulation Research, 122, 1545–1554. 29669712 10.1161/CIRCRESAHA.117.312641 PMC5970094

[phy270400-bib-0003] Biganzoli, E. , Desmedt, C. , Fornili, M. , de Azambuja, E. , Cornez, N. , Ries, F. , Closon‐Dejardin, M. T. , Kerger, J. , Focan, C. , Di Leo, A. , Nogaret, J. M. , Sotiriou, C. , Piccart, M. , & Demicheli, R. (2017). Recurrence dynamics of breast cancer according to baseline body mass index. European Journal of Cancer, 87, 10–20. 29096156 10.1016/j.ejca.2017.10.007

[phy270400-bib-0004] Carvalho, R. A. , Sousa, R. P. , Cadete, V. J. , Lopaschuk, G. D. , Palmeira, C. M. , Bjork, J. A. , & Wallace, K. B. (2010). Metabolic remodeling associated with subchronic doxorubicin cardiomyopathy. Toxicology, 270, 92–98. 20132857 10.1016/j.tox.2010.01.019

[phy270400-bib-0005] Chen, L. , Li, Q. , She, T. , Li, H. , Yue, Y. , Gao, S. , Yan, T. , Liu, S. , Ma, J. , & Wang, Y. (2016). IRE1alpha‐XBP1 signaling pathway, a potential therapeutic target in multiple myeloma. Leukemia Research, 49, 7–12. 27518808 10.1016/j.leukres.2016.07.006

[phy270400-bib-0006] Chen, X. , Iliopoulos, D. , Zhang, Q. , Tang, Q. , Greenblatt, M. B. , Hatziapostolou, M. , Lim, E. , Tam, W. L. , Ni, M. , Chen, Y. , Mai, J. , Shen, H. , Hu, D. Z. , Adoro, S. , Hu, B. , Song, M. , Tan, C. , Landis, M. D. , Ferrari, M. , … Glimcher, L. H. (2014). XBP1 promotes triple‐negative breast cancer by controlling the HIF1α pathway. Nature, 508, 103–107. 24670641 10.1038/nature13119 PMC4105133

[phy270400-bib-0007] Chen, Y. , Huang, T. , Shi, W. , Fang, J. , Deng, H. , & Cui, G. (2020). Potential targets for intervention against doxorubicin‐induced cardiotoxicity based on genetic studies: A systematic review of the literature. Journal of Molecular and Cellular Cardiology, 138, 88–98. 31751567 10.1016/j.yjmcc.2019.11.150

[phy270400-bib-0008] Chien, W. , Ding, L. W. , Sun, Q. Y. , Torres‐Fernandez, L. A. , Tan, S. Z. , Xiao, J. , Lim, S. L. , Garg, M. , Lee, K. L. , Kitajima, S. , Takao, S. , Leong, W. Z. , Sun, H. , Tokatly, I. , Poellinger, L. , Gery, S. , & Koeffler, P. H. (2014). Selective inhibition of unfolded protein response induces apoptosis in pancreatic cancer cells. Oncotarget, 5, 4881–4894. 24952679 10.18632/oncotarget.2051 PMC4148107

[phy270400-bib-0009] Clarke, R. , & Cook, K. L. (2015). Unfolding the role of stress response signaling in endocrine resistant breast cancers. Frontiers in Oncology, 5, 140. 26157705 10.3389/fonc.2015.00140 PMC4475795

[phy270400-bib-0010] Clarke, R. , Cook, K. L. , Hu, R. , Facey, C. O. , Tavassoly, I. , Schwartz, J. L. , Baumann, W. T. , Tyson, J. J. , Xuan, J. , Wang, Y. , Wärri, A. , & Shajahan, A. N. (2012). Endoplasmic reticulum stress, the unfolded protein response, autophagy, and the integrated regulation of breast cancer cell fate. Cancer Research, 72, 1321–1331. 22422988 10.1158/0008-5472.CAN-11-3213 PMC3313080

[phy270400-bib-0011] Feitelson, M. A. , Arzumanyan, A. , Kulathinal, R. J. , Blain, S. W. , Holcombe, R. F. , Mahajna, J. , Marino, M. , Martinez‐Chantar, M. L. , Nawroth, R. , Sanchez‐Garcia, I. , Sharma, D. , Saxena, N. K. , Singh, N. , Vlachostergios, P. J. , Guo, S. , Honoki, K. , Fujii, H. , Georgakilas, A. G. , Bilsland, A. , … Nowsheen, S. (2015). Sustained proliferation in cancer: Mechanisms and novel therapeutic targets. Seminars in Cancer Biology, 35(Suppl), S25–S54. 25892662 10.1016/j.semcancer.2015.02.006 PMC4898971

[phy270400-bib-0012] Feliz‐Mosquea, Y. R. , Christensen, A. A. , Wilson, A. S. , Westwood, B. , Varagic, J. , Melendez, G. C. , Schwartz, A. L. , Chen, Q. R. , Mathews Griner, L. , Guha, R. , Thomas, C. J. , Ferrer, M. , Merino, M. J. , Cook, K. L. , Roberts, D. D. , & Soto‐Pantoja, D. R. (2018). Combination of anthracyclines and anti‐CD47 therapy inhibit invasive breast cancer growth while preventing cardiac toxicity by regulation of autophagy. Breast Cancer Research and Treatment, 172, 69–82. 30056566 10.1007/s10549-018-4884-x PMC6195817

[phy270400-bib-0013] Foulkes, W. D. , Smith, I. E. , & Reis‐Filho, J. S. (2010). Triple‐negative breast cancer. The New England Journal of Medicine, 363, 1938–1948. 21067385 10.1056/NEJMra1001389

[phy270400-bib-0014] Friedrich, M. J. (2017). Global obesity epidemic worsening. JAMA, 318, 603. 10.1001/jama.2017.10693 28810033

[phy270400-bib-0015] Fu, F. , & Doroudgar, S. (2022). IRE1/XBP1 and endoplasmic reticulum signaling—From basic to translational research for cardiovascular disease. Current Opinion in Physiology, 28, 100552. 37207249 10.1016/j.cophys.2022.100552 PMC10195104

[phy270400-bib-0016] Glembotski, C. C. (2007). Endoplasmic reticulum stress in the heart. Circulation Research, 101, 975–984. 17991891 10.1161/CIRCRESAHA.107.161273

[phy270400-bib-0017] Goormaghtigh, E. , Pollakis, G. , & Ruysschaert, J. M. (1983). Mitochondrial membrane modifications induced by adriamycin‐mediated electron transport. Biochemical Pharmacology, 32, 889–893. 6838634 10.1016/0006-2952(83)90593-2

[phy270400-bib-0018] Hamada, H. , Suzuki, M. , Yuasa, S. , Mimura, N. , Shinozuka, N. , Takada, Y. , Suzuki, M. , Nishino, T. , Nakaya, H. , Koseki, H. , & Aoe, T. (2004). Dilated cardiomyopathy caused by aberrant endoplasmic reticulum quality control in mutant KDEL receptor transgenic mice. Molecular and Cellular Biology, 24, 8007–8017. 15340063 10.1128/MCB.24.18.8007-8017.2004 PMC515036

[phy270400-bib-0019] Hong, J. , Kim, K. , Kim, J. H. , & Park, Y. (2017). The role of endoplasmic reticulum stress in cardiovascular disease and exercise. International Journal of Vascular Medicine, 2017, 2049217. 28875043 10.1155/2017/2049217 PMC5569752

[phy270400-bib-0020] Hu, R. , Warri, A. , Jin, L. , Zwart, A. , Riggins, R. B. , Fang, H. B. , & Clarke, R. (2015). NF‐kappaB signaling is required for XBP1 (unspliced and spliced)‐mediated effects on antiestrogen responsiveness and cell fate decisions in breast cancer. Molecular and Cellular Biology, 35, 379–390. 25368386 10.1128/MCB.00847-14 PMC4272419

[phy270400-bib-0021] Kashfi, K. , Israel, M. , Sweatman, T. W. , Seshadri, R. , & Cook, G. A. (1990). Inhibition of mitochondrial carnitine palmitoyltransferases by adriamycin and adriamycin analogues. Biochemical Pharmacology, 40, 1441–1448. 2222502 10.1016/0006-2952(90)90438-q

[phy270400-bib-0022] Kim, D. H. (2016). IRE1 sulfenylation by reactive oxygen species coordinates cellular stress signaling. Molecular Cell, 63, 541–542. 27540852 10.1016/j.molcel.2016.08.003

[phy270400-bib-0023] Leitner, D. R. , Fruhbeck, G. , Yumuk, V. , Schindler, K. , Micic, D. , Woodward, E. , & Toplak, H. (2017). Obesity and type 2 diabetes: Two diseases with a need for combined treatment strategies—EASO can lead the way. Obesity Facts, 10, 483–492. 29020674 10.1159/000480525 PMC5741209

[phy270400-bib-0024] Lin, J. H. , Walter, P. , & Yen, T. S. (2008). Endoplasmic reticulum stress in disease pathogenesis. Annual Review of Pathology, 3, 399–425. 10.1146/annurev.pathmechdis.3.121806.151434 PMC3653419 18039139

[phy270400-bib-0025] Liu, X. H. , Zhang, Z. Y. , Sun, S. , & Wu, X. D. (2008). Ischemic postconditioning protects myocardium from ischemia/reperfusion injury through attenuating endoplasmic reticulum stress. Shock, 30, 422–427. 18323739 10.1097/SHK.0b013e318164ca29

[phy270400-bib-0026] Logue, S. E. , McGrath, E. P. , Cleary, P. , Greene, S. , Mnich, K. , Almanza, A. , Chevet, E. , Dwyer, R. M. , Oommen, A. , Legembre, P. , Godey, F. , Madden, E. C. , Leuzzi, B. , Obacz, J. , Zeng, Q. , Patterson, J. B. , Jager, R. , Gorman, A. M. , & Samali, A. (2018). Inhibition of IRE1 RNase activity modulates the tumor cell secretome and enhances response to chemotherapy. Nature Communications, 9, 3267. 10.1038/s41467-018-05763-8 PMC6093931 30111846

[phy270400-bib-0027] Marek‐Iannucci, S. , Yildirim, A. D. , Hamid, S. M. , Ozdemir, A. B. , Gomez, A. C. , Kocaturk, B. , Porritt, R. A. , Fishbein, M. C. , Iwawaki, T. , Noval Rivas, M. , Erbay, E. , & Arditi, M. (2022). Targeting IRE1 endoribonuclease activity alleviates cardiovascular lesions in a murine model of Kawasaki disease vasculitis. JCI Insight, 7, e157203. 35167493 10.1172/jci.insight.157203 PMC8986066

[phy270400-bib-0028] Martindale, J. J. , Fernandez, R. , Thuerauf, D. , Whittaker, R. , Gude, N. , Sussman, M. A. , & Glembotski, C. C. (2006). Endoplasmic reticulum stress gene induction and protection from ischemia/reperfusion injury in the hearts of transgenic mice with a tamoxifen‐regulated form of ATF6. Circulation Research, 98, 1186–1193. 16601230 10.1161/01.RES.0000220643.65941.8d

[phy270400-bib-0029] Minamino, T. , & Kitakaze, M. (2010). ER stress in cardiovascular disease. Journal of Molecular and Cellular Cardiology, 48, 1105–1110. 19913545 10.1016/j.yjmcc.2009.10.026

[phy270400-bib-0030] Ming, J. , Ruan, S. , Wang, M. , Ye, D. , Fan, N. , Meng, Q. , Tian, B. , & Huang, T. (2015). A novel chemical, STF‐083010, reverses tamoxifen‐related drug resistance in breast cancer by inhibiting IRE1/XBP1. Oncotarget, 6, 40692–40703. 26517687 10.18632/oncotarget.5827 PMC4747362

[phy270400-bib-0031] Ng, M. , Fleming, T. , Robinson, M. , Thomson, B. , Graetz, N. , Margono, C. , Mullany, E. C. , Biryukov, S. , Abbafati, C. , Abera, S. F. , Abraham, J. P. , Abu‐Rmeileh, N. M. , Achoki, T. , AlBuhairan, F. S. , Alemu, Z. A. , Alfonso, R. , Ali, M. K. , Ali, R. , Guzman, N. A. , … Gakidou, E. (2014). Global, regional, and national prevalence of overweight and obesity in children and adults during 1980‐2013: A systematic analysis for the global burden of disease study 2013. Lancet (London, England), 384, 766–781. 24880830 10.1016/S0140-6736(14)60460-8 PMC4624264

[phy270400-bib-0032] Okada, K. , Minamino, T. , Tsukamoto, Y. , Liao, Y. , Tsukamoto, O. , Takashima, S. , Hirata, A. , Fujita, M. , Nagamachi, Y. , Nakatani, T. , Yutani, C. , Ozawa, K. , Ogawa, S. , Tomoike, H. , Hori, M. , & Kitakaze, M. (2004). Prolonged endoplasmic reticulum stress in hypertrophic and failing heart after aortic constriction: Possible contribution of endoplasmic reticulum stress to cardiac myocyte apoptosis. Circulation, 110, 705–712. 15289376 10.1161/01.CIR.0000137836.95625.D4

[phy270400-bib-0033] Ortega, A. , Rosello‐Lleti, E. , Tarazon, E. , Molina‐Navarro, M. M. , Martinez‐Dolz, L. , Gonzalez‐Juanatey, J. R. , Lago, F. , Montoro‐Mateos, J. D. , Salvador, A. , Rivera, M. , & Portoles, M. (2014). Endoplasmic reticulum stress induces different molecular structural alterations in human dilated and ischemic cardiomyopathy. PLoS One, 9, e107635. 25226522 10.1371/journal.pone.0107635 PMC4166610

[phy270400-bib-0034] Petrovski, G. , Das, S. , Juhasz, B. , Kertesz, A. , Tosaki, A. , & Das, D. K. (2011). Cardioprotection by endoplasmic reticulum stress‐induced autophagy. Antioxidants & Redox Signaling, 14, 2191–2200. 20726815 10.1089/ars.2010.3486

[phy270400-bib-0035] Pierobon, M. , & Frankenfeld, C. L. (2013). Obesity as a risk factor for triple‐negative breast cancers: A systematic review and meta‐analysis. Breast Cancer Research and Treatment, 137, 307–314. 23179600 10.1007/s10549-012-2339-3

[phy270400-bib-0036] Protani, M. , Coory, M. , & Martin, J. H. (2010). Effect of obesity on survival of women with breast cancer: Systematic review and meta‐analysis. Breast Cancer Research and Treatment, 123, 627–635. 20571870 10.1007/s10549-010-0990-0

[phy270400-bib-0037] Qiu, Y. , Jiang, P. , & Huang, Y. (2023). Anthracycline‐induced cardiotoxicity: Mechanisms, monitoring, and prevention. Frontiers in Cardiovascular Medicine, 10, 1242596. 38173817 10.3389/fcvm.2023.1242596 PMC10762801

[phy270400-bib-0038] Qu, J. , Li, M. , Li, D. , Xin, Y. , Li, J. , Lei, S. , Wu, W. , & Liu, X. (2021). Stimulation of sigma‐1 receptor protects against cardiac fibrosis by alleviating IRE1 pathway and autophagy impairment. Oxidative Medicine and Cellular Longevity, 2021, 8836818. 33488945 10.1155/2021/8836818 PMC7801073

[phy270400-bib-0039] Raj, S. , Franco, V. I. , & Lipshultz, S. E. (2014). Anthracycline‐induced cardiotoxicity: A review of pathophysiology, diagnosis, and treatment. Current Treatment Options in Cardiovascular Medicine, 16, 315. 24748018 10.1007/s11936-014-0315-4

[phy270400-bib-0040] Ramirez, M. U. , Clear, K. Y. J. , Cornelius, Z. , Bawaneh, A. , Feliz‐Mosquea, Y. R. , Wilson, A. S. , Ruggiero, A. D. , Cruz‐Diaz, N. , Shi, L. , Kerr, B. A. , Soto‐Pantoja, D. R. , & Cook, K. L. (2022). Diet impacts triple‐negative breast cancer growth, metastatic potential, chemotherapy responsiveness, and doxorubicin‐mediated cardiac dysfunction. Physiological Reports, 10, e15192. 35439354 10.14814/phy2.15192 PMC9017973

[phy270400-bib-0041] Schlame, M. , Rua, D. , & Greenberg, M. L. (2000). The biosynthesis and functional role of cardiolipin. Progress in Lipid Research, 39, 257–288. 10799718 10.1016/s0163-7827(00)00005-9

[phy270400-bib-0042] Siegel, R. L. , Giaquinto, A. N. , & Jemal, A. (2024). Cancer statistics, 2024. CA: A Cancer Journal for Clinicians, 74, 12–49. 38230766 10.3322/caac.21820

[phy270400-bib-0043] Sikov, W. M. , Berry, D. A. , Perou, C. M. , Singh, B. , Cirrincione, C. T. , Tolaney, S. M. , Kuzma, C. S. , Pluard, T. J. , Somlo, G. , Port, E. R. , Golshan, M. , Bellon, J. R. , Collyar, D. , Hahn, O. M. , Carey, L. A. , Hudis, C. A. , & Winer, E. P. (2015). Impact of the addition of carboplatin and/or bevacizumab to neoadjuvant once‐per‐week paclitaxel followed by dose‐dense doxorubicin and cyclophosphamide on pathologic complete response rates in stage II to III triple‐negative breast cancer: CALGB 40603 (Alliance). Journal of Clinical Oncology: Official Journal of the American Society of Clinical Oncology, 33, 13–21. 25092775 10.1200/JCO.2014.57.0572 PMC4268249

[phy270400-bib-0044] Sorlie, T. , Tibshirani, R. , Parker, J. , Hastie, T. , Marron, J. S. , Nobel, A. , Deng, S. , Johnsen, H. , Pesich, R. , Geisler, S. , Demeter, J. , Perou, C. M. , Lønning, P. E. , Brown, P. O. , Børresen‐Dale, A. L. , & Botstein, D. (2003). Repeated observation of breast tumor subtypes in independent gene expression data sets. Proceedings of the National Academy of Sciences of the United States of America, 100, 8418–8423. 12829800 10.1073/pnas.0932692100 PMC166244

[phy270400-bib-0045] Soto‐Pantoja, D. R. , Wilson, A. S. , Clear, K. Y. , Westwood, B. , Triozzi, P. L. , & Cook, K. L. (2017). Unfolded protein response signaling impacts macrophage polarity to modulate breast cancer cell clearance and melanoma immune checkpoint therapy responsiveness. Oncotarget, 8, 80545–80559. 29113324 10.18632/oncotarget.19849 PMC5655219

[phy270400-bib-0046] Steinhorn, B. , Sorrentino, A. , Badole, S. , Bogdanova, Y. , Belousov, V. , & Michel, T. (2018). Chemogenetic generation of hydrogen peroxide in the heart induces severe cardiac dysfunction. Nature Communications, 9, 4044. 10.1038/s41467-018-06533-2 PMC6168530 30279532

[phy270400-bib-0047] Sun, H. , Zou, J. , Chen, L. , Zu, X. , Wen, G. , & Zhong, J. (2017). Triple‐negative breast cancer and its association with obesity. Molecular and Clinical Oncology, 7, 935–942. 29285353 10.3892/mco.2017.1429 PMC5740844

[phy270400-bib-0048] Suraweera, C. D. , Hinds, M. G. , & Kvansakul, M. (2020). Poxviral strategies to overcome host cell apoptosis. Pathogens, 10, 6. 33374867 10.3390/pathogens10010006 PMC7823800

[phy270400-bib-0049] Suzuki, R. , Orsini, N. , Saji, S. , Key, T. J. , & Wolk, A. (2009). Body weight and incidence of breast cancer defined by estrogen and progesterone receptor status—A meta‐analysis. International Journal of Cancer, 124, 698–712. 18988226 10.1002/ijc.23943

[phy270400-bib-0050] Thuerauf, D. J. , Marcinko, M. , Gude, N. , Rubio, M. , Sussman, M. A. , & Glembotski, C. C. (2006). Activation of the unfolded protein response in infarcted mouse heart and hypoxic cultured cardiac myocytes. Circulation Research, 99, 275–282. 16794188 10.1161/01.RES.0000233317.70421.03

[phy270400-bib-0051] Uemura, A. , Oku, M. , Mori, K. , & Yoshida, H. (2009). Unconventional splicing of XBP1 mRNA occurs in the cytoplasm during the mammalian unfolded protein response. Journal of Cell Science, 122, 2877–2886. 19622636 10.1242/jcs.040584

[phy270400-bib-0052] Ventura‐Clapier, R. , Garnier, A. , & Veksler, V. (2004). Energy metabolism in heart failure. The Journal of Physiology, 555, 1–13. 14660709 10.1113/jphysiol.2003.055095 PMC1664831

[phy270400-bib-0053] Wang, Q. , Zhao, Y. , Zheng, H. , Wang, Q. , Wang, W. , Liu, B. , Han, H. , Zhang, L. , & Chen, K. (2020). CCDC170 affects breast cancer apoptosis through IRE1 pathway. Aging (Albany NY), 13, 1332–1356. 33291081 10.18632/aging.202315 PMC7835043

[phy270400-bib-0054] Wang, S. , Binder, P. , Fang, Q. , Wang, Z. , Xiao, W. , Liu, W. , & Wang, X. (2018). Endoplasmic reticulum stress in the heart: Insights into mechanisms and drug targets. British Journal of Pharmacology, 175, 1293–1304. 28548229 10.1111/bph.13888 PMC5867005

[phy270400-bib-0055] Wang, S. , & Kaufman, R. J. (2012). The impact of the unfolded protein response on human disease. The Journal of Cell Biology, 197, 857–867. 22733998 10.1083/jcb.201110131 PMC3384412

[phy270400-bib-0056] Yang, S. , Zhang, J. J. , & Huang, X. Y. (2012). Mouse models for tumor metastasis. Methods in Molecular Biology, 928, 221–228. 22956145 10.1007/978-1-62703-008-3_17 PMC3674868

[phy270400-bib-0057] Zhao, N. , Cao, J. , Xu, L. , Tang, Q. , Dobrolecki, L. E. , Lv, X. , Talukdar, M. , Lu, Y. , Wang, X. , Hu, D. Z. , Shi, Q. , Xiang, Y. , Wang, Y. , Liu, X. , Bu, W. , Jiang, Y. , Li, M. , Gong, Y. , Sun, Z. , … Chen, X. (2018). Pharmacological targeting of MYC‐regulated IRE1/XBP1 pathway suppresses MYC‐driven breast cancer. The Journal of Clinical Investigation, 128, 1283–1299. 29480818 10.1172/JCI95873 PMC5873887

[phy270400-bib-0058] Zhu, H. , Sarkar, S. , Scott, L. , Danelisen, I. , Trush, M. A. , Jia, Z. , & Li, Y. R. (2016). Doxorubicin redox biology: Redox cycling, topoisomerase inhibition, and oxidative stress. React Oxyg Species (Apex), 1, 189–198. 29707645 10.20455/ros.2016.835 PMC5921833

