# Supplementary material for: Dietary influences on chemotherapy sensitivity and cardiotoxicity modulated by IRE1 targeting in triple‐negative breast cancer in female mice
Source: Physiol Rep. 2025 Sep 21;13(18):e70400. doi: 10.14814/phy2.70400 (PMC12451018; doi:10.14814/phy2.70400)
Supplement: Supplementary file 1 — Data S1. [file PHY2-13-e70400-s001.pdf]

Supplemental Figure S1

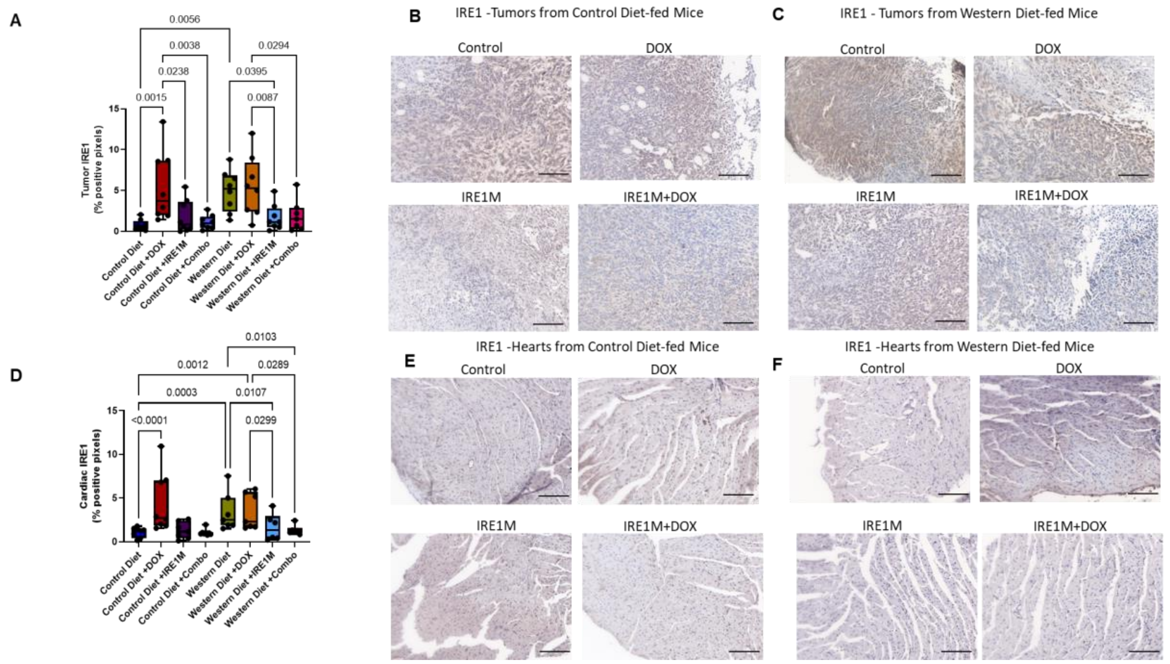

Supplemental Figure S1. IRE1 morpholino reduces tumor and heart IRE1 protein levels. IRE1 mouse anti-sense morpholino reduces IRE1 protein levels in tumors and hearts. **A-F.** Tumor and cardiac tissue were obtained from control and Western diet-fed mice, paraffin-embedded and sectioned. Sections of tumors **(A)** and hearts **(D)** were stained with DAB (IRE1α). n = 7-8; analyzed by two-way ANOVA followed by a Tukey's multiple comparison test. Representative image of IRE1α staining 4T1 tumors **(B-C)** and hearts **(E-F)** from control and Western-fed mice. IRE1 (Inositol-requiring enzyme-1), DAB (3,3'-Diaminobenzidine). Scale bar = 100 µm.

Supplemental Figure S2

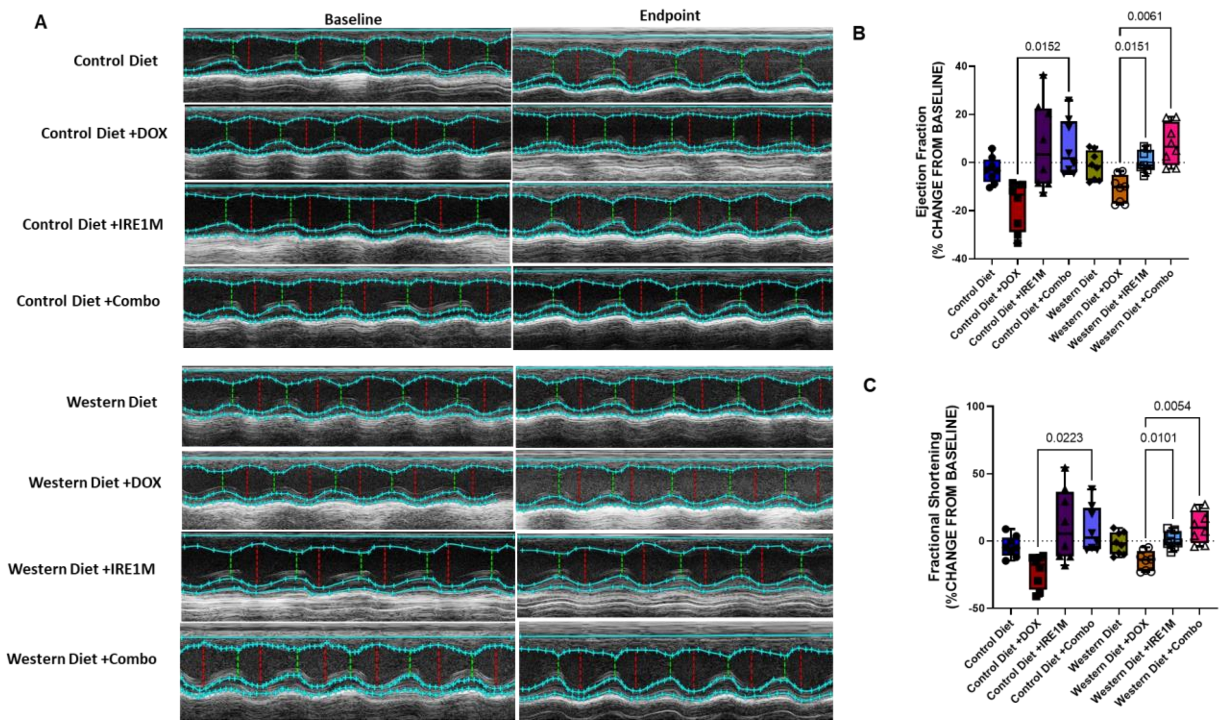

Supplemental Figure S2. IRE1 blockade modules cardiac dysfunction. A. Representative image of M-mode tracing using Vevo LAZR ultrasound in mice consuming control and Western diets at baseline and after DOX chemotherapy. DOX (Doxorubicin). **B.** Ejection fraction in mice consuming control and Western diets at baseline, bearing primary 4T1 breast tumor, after injected I.V. with DOX (endpoint), graphed as percent change from baseline n=8. Analyzed by Brown-Forsythe and Welch ANOVA tests followed by Tukey's multiple comparison test. **C.** Fractional shortening in mice consuming control and Western diets at baseline, bearing primary 4T1 breast tumor, after injected I.V. with DOX (endpoint), graphed as percent change from baseline n=8; analyzed by Brown-Forsythe and Welch ANOVA tests followed by Tukey's multiple comparison test.

Supplemental Figure S3

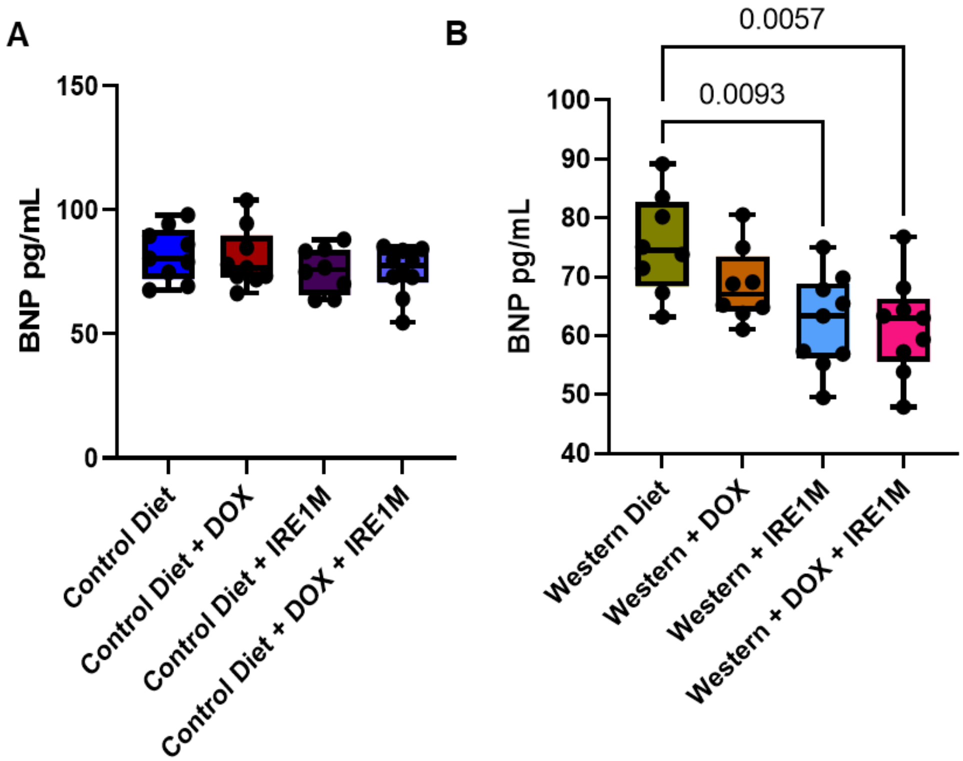

Supplemental Figure S3. Plasma cardiac damage marker BNP is not regulated by DOX. Plasma levels of the cardiac biomarker BNP (B-Type Natriuretic Peptide) of Control (A) and Western (B) diets fed mice n=8-10; analyzed by one-way ANOVA followed by Tukey's multiple comparison test.
